# Refractory hypercalcemia of malignancy: a problem with many potential roots

**DOI:** 10.3389/fendo.2023.1088984

**Published:** 2023-05-17

**Authors:** Azeez Farooki

**Affiliations:** ^1^ Endocrinology Service, Department of Medicine, Memorial Sloan Kettering Cancer Center, New York, NY, United States; ^2^ Department of Medicine, Division of Endocrinology, Diabetes, and Metabolism, NewYork-Presbyterian/Weill Cornell Medical Center, New York, NY, United States

**Keywords:** hypercalcemia of malignancy, denosumab, intravenous bisphosphonates, calcitriol, bone resorption, bone resorption and formation, hypercalciuria, PTHrP (parathyroid hormone-related protein)

## Abstract

Hypercalcemia of malignancy (HCM) is a common clinical problem that is associated with considerable morbidity and negative effects on quality of life. Despite the availability of effective medical treatments for HCM, options are needed for cases that are refractory to conventional therapies. In this context, “refractory” refers to reasonable control of calcium in the setting of inpatient hospitalization (after receipt of standard of care therapies, such as continuous intravenous fluids, calcitonin, and intravenous bisphosphonates) with relapse into severe hypercalcemia within days or weeks of discharge from the hospital. Here we discuss drivers of hypercalcemia of malignancy and the physiologic mechanisms whereby they operate to increase serum calcium. Additionally, we discuss multiple available treatments targeted to a given contributory mechanism and also briefly discuss potential future treatments in need of further study.

## 1 Introduction

Hypercalcemia of malignancy (HCM) is a common clinical problem that is associated with considerable morbidity and negative effects on quality of life. Despite the availability of effective medical treatments for HCM, options are needed for cases that are refractory to conventional therapies—this is most often seen in patients with solid tumors. In this context, “refractory” refers to reasonable control of calcium in the setting of inpatient hospitalization (after receipt of standard of care therapies, such as continuous intravenous fluids, calcitonin, and intravenous bisphosphonates) with relapse into severe hypercalcemia within days or weeks of discharge from the hospital. Inpatient readmissions may occur until, in a best-case situation, the use of antineoplastic therapy controls the tumor and the calcium level becomes manageable.

## 2 Contributory mechanisms

Multiple contributory mechanisms may lead to the development of treatment-refractory HCM. Benign primary hyperparathyroidism can coexist with HCM and should be identified as a possible contributor. It is generally excluded if the parathyroid hormone (PTH) value is <20 pg/mL (termed PTH-independent hypercalcemia). In addition to benign primary hyperparathyroidism, an elevated PTH value is present in parathyroid carcinoma and in rare cases of ectopic PTH secretion by tumors. The basic pathophysiologic mechanisms causing HCM include elevations in bone resorption, acceleration of gastrointestinal calcium absorption, and diminished renal calcium excretion. **Table 1** provides a summary of the endocrine driver(s) for a given mechanism. These mechanisms are not mutually exclusive, and on the basis of findings from our retrospective series (1), more than one mechanism may be active in a patient. For example, a common misconception is that patients with parathyroid hormone–related protein (PTHrP)–dependent HCM are unlikely to have elevated levels of calcitriol; however, such patients may have elevations in both PTHrP and calcitriol. In our retrospective series of patients with PTH-independent HCM due to solid tumors, of the 45 patients with calcitriol elevation, 34 (76%) also had PTHrP elevations (1). In cases of HCM refractory to standard potent antiresorptive therapies (intravenous bisphosphonates and denosumab), targeting one or more of these contributory mechanisms is often necessary.

**Table 1:** Endocrine drivers of mechanisms that contribute to PTH-independent refractory HCM, with specific therapies for each mechanism.

Mechanism	Driver			Therapy targeted to contributory mechanism
	Endocrine		Other	
	Inappropriate calcitriol production	PTHrP production	Local osteolysis (advanced cancer in the bone)	
Bone resorption	+	+++	+++	Intravenous bisphosphonates, denosumab
Gastrointestinal calcium absorption	+++	*	–	Corticosteroids or other inhibitor of 1-alpha-hydroxylase; limit dietary calcium intake
Renal calcium reabsorption	–	+++	–	Frequent intravenous fluids (normal saline with furosemide); cinacalcet
Hypophosphatemia (less CaPO4 = increased serum calcium)	–	++	–	Oral phosphorus

Note that corticosteroids and phosphorus may be helpful regardless of the causative etiology. All mechanisms listed may be active in a given patient. For example, a patient with refractory hypercalcemia of malignancy may have all of the following: metastatic bone disease, high PTHrP level, hypophosphatemia, elevated calcitriol level, and relative hypocalciuria (relative to the degree of hypercalcemia, a calcium-retaining effect mediated by PTHrP). + relative contribution of a given mechanism; - lack of contribution; *potential indirect effect: only if high PTHrP stimulates calcitriol production.

## 3 Treatment options

### 3.1 HCM

Inhibition of osteoclastic activity or bone resorption is a validated strategy for treatment of HCM. At present, parenteral bisphosphonates and denosumab represent the most potent antiresorptives available. These drugs decrease bone turnover via inhibition of osteoclastic activity; since the process of bone turnover (sequential osteoclast activity followed by osteoblast activity) is tightly coupled, osteoblastic activity is also decreased. Together with standard of care therapies (intravenous fluids and subcutaneous calcitonin), the use of these agents often results in resolution of HCM; however, refractory cases are not uncommon in our experience. Furthermore, particularly when parenteral bisphosphonates and denosumab are used at an oncologic dosing frequency (monthly to every 3 months), rare but significant adverse effects, such as osteonecrosis of the jaw and atypical femur fracture (2), can occur.

### 3.2 Refractory HCM

What are the treatment options for cases of refractory HCM? To begin, a laboratory workup can help reveal the pathophysiologic mechanisms involved. Before the advent of intravenous bisphosphonates and denosumab, oral and intravenous phosphorus was used therapeutically to treat HCM (3). Phosphorus binds calcium and then, in the presence of normal renal function, is eliminated (likely via the reticuloendothelial system) and also can be deposited in bone or possibly in extra skeletal tissues. Therefore, it makes good clinical sense to treat the often severe hypophosphatemia that occurs frequently in PTHrP-mediated HCM. It should also be noted that advanced cancer can rarely secrete FGF-23 and, thereby, cause severe hypophosphatemia and subsequent bone fragility via induction of osteomalacia (4). Corticosteroids work to decrease calcium likely via multiple mechanisms, including a decrease in inflammatory mediators (which otherwise act in the bone microenvironment to increase bone resorption) and inhibition of 1-alpha-hydroxylase, resulting in a decrease in intestinal calcium absorption. Steroids, which are viewed as a standard treatment for hypercalcemia associated with granulomatous diseases and lymphoma, have also long been used to treat HCM in patients with solid tumors (5).

Retrospective data from our group and other groups (Sheehan et al. https://academic.oup.com/jes/article/5/11/bvab157/6382077) has shown that elevated calcitriol in patients with solid tumors is more common than previously believed and is associated with a lack of response to antiresorptive therapy (1). The etiology of such elevations in calcitriol is unclear; there was no clear correlation with PTHrP elevation or with hypophosphatemia, respectively (1). We observed a markedly elevated odds ratio (OR) for lack of response to antiresorptive therapy in cases of elevated calcitriol, even compared with elevated PTHrP, a powerful driver of HCM (OR for calcitriol elevation, 15.22 [95% CI 5.12-52.58] [p<0.001] vs OR for PTHrP elevation, 3.05 [95% CI 0.95-10.58] [p=0.066]). In our experience, cases of refractory HCM with elevated calcitriol do respond to steroid therapy. If steroids are not able to be used, alternative inhibitors of 1-alpha-hydroxylase can be considered, as they are sometimes used in other clinical contexts where calcitriol is overproduced (6–8).

Accelerated renal tubular calcium reabsorption plays a significant role in cases of PTHrP-mediated HCM. In a well-hydrated hypercalcemic patient receiving normal saline, furosemide-induced calciuresis can be used to try to overcome the renal calcium retention induced by PTHrP; however, as the half-life of furosemide is 2 h, the beneficial effect of this approach is transient. Finally, repeated dosing of zoledronic acid weekly or denosumab once weekly can also be considered; one study showed a calcium-lowering benefit to weekly denosumab in subjects who had previously received intravenous pamidronate or zoledronic acid at the more standard monthly dosing (1, 9), which is the approved dosing to reduce the risk of skeletal-related events in patients with solid tumors. Although cases refractory to bisphosphonate and denosumab illustrate the limits of inhibition of bone resorption with these agents, an unexplored area is the manipulation of bone formation, which has been shown to be decreased by continuous PTHrP infusion in humans (10). A drug such as romosozumab, which increases bone formation while restraining bone resorption, could theoretically play a role in driving bone calcium influx to lower serum calcium.

Cases of refractory HCM that responded to cinacalcet in patients with solid tumors have been reported (11, 12). Cinacalcet may certainly be used in patients with parathyroid carcinoma in which PTH is produced inappropriately. Why would this agent be beneficial if PTH levels are nearly zero? A theoretical mechanism of benefit from cinacalcet in cases of PTHrP-mediated HCM has been articulately proposed (12). This theory holds that the action of PTHrP to conserve calcium loss is blocked by the action of cinacalcet at the calcium-sensing receptor in the distal nephron and that the drug could cause a relative increase in renal calcium excretion to help manage HCM. Thus, in PTHrP-mediated HCM, this approach could be especially useful to counteract retention of renal calcium, without the risk of volume depletion induced by the repeated use of furosemide therapy.

A PTH receptor antagonist is currently in preclinical development (13). Preliminary data show that this orally available agent was effective in a rat model of HCM induced by continuous infusion of PTH. If validated in human trials, such an agent could prove to be an effective therapy for cases of HCM refractory to bisphosphonates and denosumab or for cases with contraindications to these agents (e.g., active osteonecrosis of the jaw or incipient atypical femur fracture).

## 4 Conclusion

Refractory HCM is an understudied clinical problem with an unmet need for effective treatment options. Targeting the responsible pathophysiologic mechanisms involved is a clinically useful strategy. In some cases, this may involve the use of antiresorptive therapy, glucocorticoids, phosphorus, and possibly cinacalcet. New agents to add to our armamentarium for the treatment of refractory HCM are eagerly anticipated.

## Data availability statement

The original contributions presented in the study are included in the article/supplementary material. Further inquiries can be directed to the corresponding author.

## Author contributions

The author confirms being the sole contributor of this work and has approved it for publication.
